# Neuronal Differentiation-Related Epigenetic Regulator *ZRF1* Has Independent Prognostic Value in Neuroblastoma but Is Functionally Dispensable In Vitro

**DOI:** 10.3390/cancers13194845

**Published:** 2021-09-28

**Authors:** Carlos Jiménez, Roberta Antonelli, Marc Masanas, Aroa Soriano, Laura Devis-Jauregui, Jessica Camacho, Ainara Magdaleno, Gabriela Guillén, Raquel Hladun, Luz Jubierre, Josep Roma, David Llobet-Navas, Josep Sánchez de Toledo, Lucas Moreno, Soledad Gallego, Miguel F. Segura

**Affiliations:** 1 Group of Translational Research in Child and Adolescent Cancer, Vall d’Hebron Research Institute (VHIR), Universitat Autònoma de Barcelona (UAB), 08035 Barcelona, Spain; carlos.jimenez@vhir.org (C.J.); roberta.antonelli@vhir.org (R.A.); marc.masanas@vhir.org (M.M.); aroa.soriano@vhir.org (A.S.); ainara.magdaleno@vhir.org (A.M.); gguillen@vhebron.net (G.G.); rhladun@vhebron.net (R.H.); jubierrl@mskcc.org (L.J.); josep.roma@vhir.org (J.R.); jsanchezdetoledo@iconcologia.net (J.S.d.T.); lucas.moreno@vhebron.net (L.M.); sgallego@vhebron.net (S.G.); 2 Molecular Mechanisms and Experimental Therapy in Oncology-Oncobell Program, Bellvitge Biomedical Research Institute (IDIBELL), 08908 L’Hospitalet de Llobregat, Spain; ldevis@idibell.cat (L.D.-J.); dllobet@idibell.cat (D.L.-N.); 3 Centro de Investigación Biomédica en Red de Cáncer (CIBERONC), Instituto de Salud Carlos III, 28029 Madrid, Spain; 4 Pathology Department, Vall d’Hebron University Hospital—UAB, 08035 Barcelona, Spain; jcamacho@vhebron.net; 5 Surgery Department, Vall d’Hebron University Hospital—UAB, 08035 Barcelona, Spain; 6 Pediatric Oncology and Hematology Department, Vall d’Hebron University Hospital—UAB, 08035 Barcelona, Spain; 7 Catalan Institute of Oncology (ICO), 08908 L’Hospitalet de Llobregat, Spain

**Keywords:** *ZRF1*, neuronal differentiation, epigenetics, pediatric cancer, neuroblastoma

## Abstract

**Simple Summary:**

Neuroblastoma is the most common pediatric solid tumor occurring outside the brain, and it is thought to arise from cells that acquire errors during the normal process of embryonal development. Today, we know that embryonal development is regulated by epigenetics, a mechanism that determines which genes need to be expressed in each cell type and developmental step. Epigenetic errors, therefore, are considered contributory to the appearance and progression of tumors such as neuroblastoma. Here, we aimed at finding whether *ZRF1*, a known epigenetic regulator, could play a significant role in the aggressiveness of neuroblastoma. Our results suggest that *ZRF1* does not seem to have any relevant function in neuroblastoma cells; however, the levels of this epigenetic regulator are related to the prognostic of neuroblastoma patients and could be used to predict their progression and improve the diagnosis.

**Abstract:**

Neuroblastoma is a pediatric tumor of the peripheral nervous system that accounts for up to ~15% of all cancer-related deaths in children. Recently, it has become evident that epigenetic deregulation is a relevant event in pediatric tumors such as high-risk neuroblastomas, and a determinant for processes, such as cell differentiation blockade and sustained proliferation, which promote tumor progression and resistance to current therapies. Thus, a better understanding of epigenetic factors implicated in the aggressive behavior of neuroblastoma cells is crucial for the development of better treatments. In this study, we characterized the role of *ZRF1*, an epigenetic activator recruited to genes involved in the maintenance of the identity of neural progenitors. We combined analysis of patient sample expression datasets with loss- and gain-of-function studies on neuroblastoma cell lines. Functional analyses revealed that *ZRF1* is functionally dispensable for those cellular functions related to cell differentiation, proliferation, migration, and invasion, and does not affect the cellular response to chemotherapeutic agents. However, we found that high levels of *ZRF1* mRNA expression are associated to shorter overall survival of neuroblastoma patients, even when those patients with the most common molecular alterations used as prognostic factors are removed from the analyses, thereby suggesting that *ZRF1* expression could be used as an independent prognostic factor in neuroblastoma.

## 1. Introduction

Neuroblastoma, a pediatric cancer of the peripheral nervous system, is one of the most common embryonal tumors outside the brain [1]. It is thought to arise from tissues of the sympathoadrenergic system at early stages of embryonic development, specifically from neural crest progenitors that fail to differentiate during dorsolateral migration [2]. From a histopathological perspective, neuroblastoma can be classified based on the grade of morphological differentiation into distinct categories, namely neuroblastoma, ganglioneuroblastoma, and ganglioneuroma, from less to more differentiated tumors. The last two categories are considered to be benign forms of neuroblastoma [3]. The pathological category can be further classified according to the degree of cellular differentiation. For example, neuroblastoma can be further classified into undifferentiated, poorly differentiated, and differentiating tumors (reviewed in [4]). The degree of differentiation is part of routine prognostic risk assessment. In fact, high-risk neuroblastomas, which represent up to ~60% of all diagnosed neuroblastomas, are usually undifferentiated or poorly differentiated tumors that present very aggressive behavior and have a five-year overall survival rate below 40% [5]. Pro-differentiation therapies (i.e., with retinoic acid derivatives) are part of the standard of care in neuroblastoma, although they are restricted to the treatment of neuroblastoma minimal residual disease [6]. However, not all patients respond to this treatment. Therefore, a better understanding of the molecular mechanisms that maintain these types of tumors in an undifferentiated state may reveal new opportunities for therapeutic intervention.

Epigenetic regulation (e.g., DNA methylation, histone post-translational modifications, non-coding RNA expression, etc.) is one of the mechanisms that controls the differentiation of neuroblasts (i.e., neuroblastoma precursors). The disruption of the homeostatic epigenetic balance contributes to the developmental arrest of sympathetic progenitors, thereby contributing to neuroblastoma oncogenesis [7]. One of the genes that is determinant in the maintenance of neuronal progenitor identity is zuotin-related factor 1 (*ZRF1*; also known as MPP11), which is encoded by the *DNAJC2* gene [8,9]. *ZRF1* belongs to the M-phase phosphoprotein (MPP) family and was first discovered as a chaperone in the cytosol [10,11,12]. However, later evidence has shown that it also acts as a chromatin regulator in the nucleus, where it is recruited to ubiquitinated histone H2A at ‘Lys-119’ (H2AK119ub), displacing the polycomb repressor complex 1 (PRC1) from chromatin and facilitating the transcription of neural progenitor-associated genes [13].

In addition to its physiological role in the maintenance of the pluripotency of neural progenitor cells, *ZRF1* has already been functionally implicated in cancer. *ZRF1* was shown to be oncogenic in solid tumors such as breast [14] and gastric [15] cancers. Furthermore, *ZRF1* was shown to be overexpressed in acute myeloid leukemia (AML) acting as a negative regulator of differentiation. In the same study, Demajo and collaborators showed that *ZRF1* depletion cooperated with differentiating agents (i.e., retinoic acid) to suppress leukemia in vivo [16].

Thus, owing to the role of *ZRF1* of maintaining the undifferentiated state of neural progenitor cells and its functional relevance in the differentiation of some tumors, we sought to determine whether *ZRF1* plays a major role in neuroblastoma. Here, we found that the expression of *ZRF1* mRNA is increased in advanced disease stages and in tumors, with the most common genetic alterations associated with poor prognosis in neuroblastoma, such as MYCN amplification, gain of chromosome 17q, and loss of 1p36. Moreover, the *ZRF1* mRNA level was clearly associated with poor neuroblastoma survival in the absence of other poor-prognosis molecular alterations. However, our gain- and loss-of-function experiments suggest that *ZRF1* is neither sufficient nor necessary to sustain the oncogenic properties of neuroblastoma cells, such as cell differentiation, proliferation, or migration. Our results validate *ZRF1* as a potential prognostic marker, but discard it as a target for differentiation therapy.

## 2. Materials and Methods

### 2.1. Analysis of Neuroblastoma Gene Expression Datasets

*ZRF1* mRNA expression levels were analyzed from neuroblastoma patient data from the GSE62564, GSE45547 and GSE3960 publicly available datasets. The GSE62564 dataset was used to construct receiver operating characteristic (ROC) curves to determine the prognostic value of *ZRF1* expression. The optimal cutoff value was defined according to the Youden index. Overall survival (OS) and the cumulative survival rate were estimated using the Kaplan–Meier method, and the log-rank test was performed to assess differences between groups. Univariate and multivariate Cox proportional hazard regression analyses were used to assess the prognostic significance of *ZRF1* on OS. These statistical analyses were performed using the IBM SPSS 21 software. For GSE45547 and GSE3960 datasets, gene expression data was extracted and Kaplan–Meier survival plots were generated using the R2 Genomics Analysis and Visualization Platform (http://r2.amc.nl; accessed date: 21 January 2021). *ZRF1* mRNA expression levels between different patient groups were analyzed using GraphPad Prism Software (La Jolla, CA, USA), and statistical significance was assessed by Kruskal–Wallis test as a non-parametric ANOVA, and Dunn’s test for multiple comparisons.

### 2.2. Cell Lines

Neuroblastoma cell lines (SK-N-AS, SH-SY5Y, and IMR-32) and embryonic kidney cells (HEK293T) were purchased from American Type Culture Collection (ATCC, Manassas, VA, USA), the CHLA-90 cell line was purchased from the Children’s Oncology Group Cell Culture and Xenograft Repository (Lubbock, TX, USA), and SK-N-BE(2), and LA1-5s were procured from the Public Health England Culture Collection (Salisbury, UK). Neuroblastoma cells were cultured and maintained in Iscove’s modified Dulbecco’s medium (Life Technologies, Waltham, MA, USA), supplemented with 20% heat-inactivated fetal bovine serum (South America Premium, Biowest, Nuaillé, France) and 1% insulin-transferrin-selenium supplement (Life Technologies). HEK293T cells were grown in Dulbecco’s modified Eagle’s medium (Life Technologies), supplemented with 10% heat-inactivated fetal bovine serum. Media were supplemented with 100 U/mL penicillin, 100 μg/mL streptomycin (Life Technologies), and 5 μg/mL plasmocin (InvivoGen, San Diego, CA, USA). Cultures were maintained at 37 °C in a 5% CO_2_ saturated atmosphere, and periodically tested for mycoplasma contamination.

### 2.3. Western Blot Analysis

Cells were lysed in RIPA buffer (Thermo Fisher Scientific, Waltham, MA, USA), supplemented with EDTA-free protease inhibitor cocktail (Roche, Basel, Switzerland). Cell lysates were quantified using a DC protein assay (Bio-Rad, Hercules, CA, USA) and 30 μg of protein was resolved on a 4–12% Tris-glycine sodium dodecyl sulfate polyacrylamide electrophoresis gel (Invitrogen, Carlsbad, CA, USA), then transferred onto polyvinylidene difluoride (PVDF) membranes. Membranes were blocked for 1 h with 5% non-fat milk or 5% bovine serum albumin (BSA) in Tris-buffered saline with 0.1% Tween and probed with primary antibodies overnight at 4 °C. Membranes were incubated with secondary antibodies for 1 h before developing with a chemiluminescent horseradish peroxidase substrate EZ-ECL Chemoluminiscence Detection Kit (Biological Industries, Kibbutz Beit-Haemek, Israel). Protein levels were quantified by densitometry using ImageJ software (National Institutes of Health, Bethesda, MD, USA). Antibodies used for Western blot are listed in Table S1. Original western blot images can be found in Figure S7.

### 2.4. Proliferation and Colony Formation Assays

For the proliferation experiments, transduced or transfected cells were plated at a density of 2–8 × 10^4^ cells /well in 6-well plates and allowed to grow for 7 days with a medium change on day 4. Cells were fixed with 1% glutaraldehyde and stained with 0.5% crystal violet. Stained cells were treated in 15% acetic acid, and the absorbance was read at 590 nm. For the colony formation experiments, cells were plated at a very low density (5–10 × 10^2^ cells/well in 6-well plates) and the medium was changed every 3–4 days. The plates were fixed in glutaraldehyde and stained with crystal violet at day 10 or when colonies were visible to the naked eye. Colonies were photographed and counted using ImageJ software.

### 2.5. Migration and Invasion Assays

For the wound-healing assays, neuroblastoma cells were plated at a density of 3 × 10^6^ cells/well in a 6-well plate. The next day, an artificial wound was created in the confluent cell monolayer. Six predefined fields per condition were photographed under contrast phase microscopy at the indicated time points, and the wound area was measured using Image J software. The migration rate was calculated by normalizing the wound area to time 0. For the transwell invasion assays, 2 × 10^5^ cells were seeded in serum-free media in the upper chamber of 8.0 µm pore size transwells (Corning Life Sciences, Corning, NY, USA) previously coated with a barrier of rat tail collagen I (Corning). The lower chamber was filled with media supplemented with fetal bovine serum. After 16 h, remaining cells were removed from the upper chamber and the cells that migrated to the lower surface of the membrane were fixed with 4% paraformaldehyde, and stained with crystal violet. Invading cells were imaged by bright field microscopy, quantified by diluting crystals in acetic acid, and read at 590 nm.

### 2.6. Differentiation Assays

Neuroblastoma cells were plated at low density (1–1.2 × 10^5^ cells) in collagen-coated 60 mm plates. One day later, cells were treated with 10 µM 13-*cis*-retinoic acid (Selleckchem, Munich, Germany). Cells were collected at day 5 post-treatment for Western blot analysis. For *RARβ* gene expression analysis, RNA was extracted from cell lysates using a miRNeasy Mini Kit (Qiagen, Germantown, MA, USA) and retrotranscribed with a high-capacity cDNA reverse transcription kit (Thermo Fisher Scientific). Real-time PCR was performed with PerfeCTa SYBR Green Fastmix (Quantabio, Beverly, MA, USA) using L27 as the internal standard. Primers are listed in Table S2. Relative quantification of gene expression was calculated using the 2^−DDCt^ method [17]. For neurite length analyses, 1× 10^4^ cells per well were seeded in collagen-coated glass covers in 24-well plates and treated with retinoic acid for 5 days before fixation with 4% paraformaldehyde. Cells were stained with phalloidin-iFluor 594 (Abcam, Cambridge, UK), following manufacturer’s instructions, and DAPI 10 µ/mL (Invitrogen). Slides were visualized with a FV1000 confocal microscope (Olympus, Shinjuku, Tokyo, Japan). Ten fields were acquired for each biological replicate and processed using ImageJ software. Actin prolongations longer than twice the length of the nucleus (~30 µm) were considered as neurites. 

### 2.7. Vectors and Lentiviral Infection

pEV-*ZRF1*, pCAG-*ZRF1*, and pLKO with different sh*ZRF1* vectors were kindly provided by Luciano DiCroce. Lentiviruses were generated in HEK293T cells using previously described methods [18,19]. Silent mutations were introduced into the *ZRF1* overexpression vector using three sequential site-directed mutagenesis reactions in pCAG-*ZRF1*, using the QuikChange II XL Site-Directed Mutagenesis Kit (Agilent, Santa Clara, CA, USA) and checked by Sanger sequencing. Primers used for site-directed mutagenesis and sequencing are listed in Table S2, and the shRNA target sequences are listed in Table S3. After mutagenesis, the *ZRF1* sequence was excised from the pCAG by XhoI digestion and ligated into the FG12 lentiviral overexpression vector.

### 2.8. ZRF1 Overexpression Experiments

Neuroblastoma cells plated at a density of 5 × 10^5^ cells in 60 mm plates were transduced with viral supernatant. pEV-transduced cells were selected by separating green fluorescence protein (GFP)-positive cells by fluorescence-assisted cell sorting (FACSAria, BD Biosciences, San Jose, CA, USA) at the Flow Cytometry facility of VHIR.

### 2.9. ZRF1 Knockdown Experiments

For shRNA transduction, 2–8 × 10^5^ cells were plated in 60 mm plates with viral supernatant containing either pLKO-non-silencing control (NSC) or shZRF #1, #2, or #3. After 16 h, the supernatant was replaced with fresh medium, and 24 h later, transduced cells were selected by puromycin resistance (1 µg/mL). Three days after transduction, the cells were detached and used for proliferation experiments. For siRNA knockdown, a set of four pre-designed ON-TARGETplus siRNAs against *ZRF1* were purchased from Dharmacon (Lafayette, CO, USA). The siRNA target sequences are listed in Table S3. Neuroblastoma cell lines at a concentration of 1.67 × 10^5^ cells/mL were transfected with siRNA at 25 nM using Lipofectamine 2000 (Life Technologies) following the manufacturer’s instructions. After incubation for 16 h, the medium was replaced.

## 3. Results

### 3.1. ZRF1 Is an Independent Prognostic Factor in Neuroblastoma

**Figure 1:**
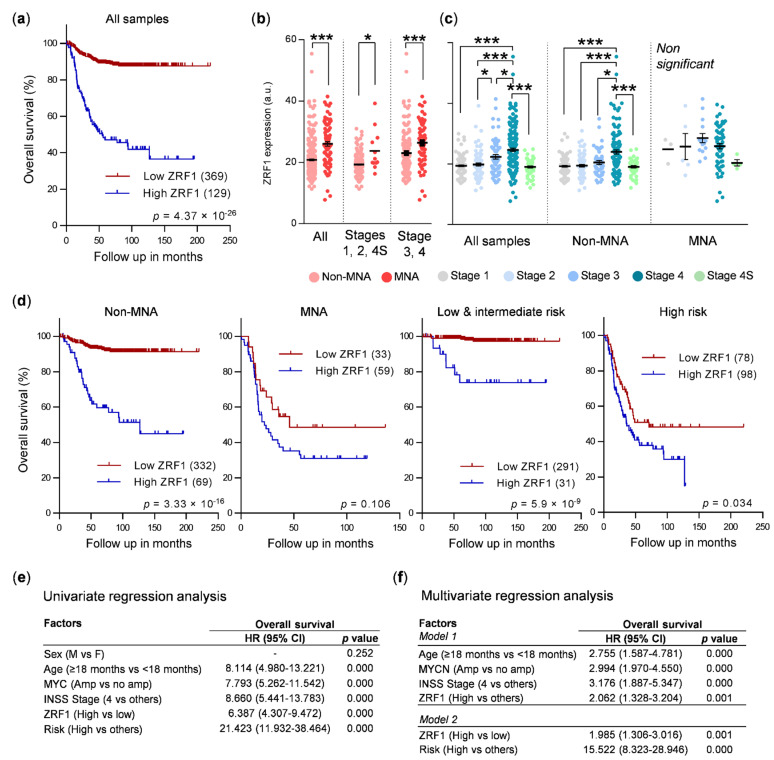
*ZRF1* is an independent prognostic factor in neuroblastoma. (**a**) Kaplan–Meier survival plot of a cohort of 498 patients (GSE62564) split into high and low *ZRF1* mRNA expression, based on the Youden index. (**b**) *ZRF1* mRNA levels in MYCN-amplified tumors (MNA, dark red) vs non-MYCN amplified tumors (non-MNA, light red), according to the indicated disease stages. (**c**) *ZRF1* mRNA levels according to disease stage in the whole cohort (left) or considering patients with non-MNA (middle) or with MNA tumors (right). (**d**) Kaplan–Meier survival plots comparing samples with high and low *ZRF1* levels in the indicated groups of patients. (**e**) Cox univariate regression analysis of overall survival with different clinic-pathological features. (**f**) Cox multivariate regression analysis of overall survival results confirm *ZRF1* mRNA levels as an independent prognostic marker in neuroblastoma. * means *p* < 0.05; *** means *p* < 0.001. HR: hazard ratio.

To determine whether *ZRF1* is involved in the biology of neuroblastoma, we analyzed publicly available mRNA expression datasets to search for correlations between *ZRF1* mRNA levels and different clinicopathological parameters of neuroblastoma patients. A receiver operating characteristic (ROC) curve analysis of a cohort of 498 patients was performed in order to assess a *ZRF1* cut-off value (Youden index) that maximized the capacity for overall survival prediction (Table S4). Using this cut-off, patients with higher *ZRF1* levels showed a reduction in overall survival when compared to patients with low *ZRF1* levels (Figure 1a). *ZRF1* was found to be upregulated in MYCN-amplified (MNA) patients, even when patients were split into early (1, 2, 4S) or advanced (3, 4) stages (Figure 1b). *ZRF1* levels were also found to be increased in advanced stages of the disease, and this upregulation was maintained even when MNA tumors were excluded (Figure 1c).

To verify whether the expression of *ZRF1* has prognostic value independent of MYCN amplification or disease stage (both factors intrinsically associated with poor survival), correlation analyses were performed excluding the MNA samples and in the different stages. The results showed that the association between *ZRF1* mRNA expression and poor survival remained in non-MNA and low stage tumors (Figure 1d). These findings were validated by contingency analyses (Table S5) and confirmed with two different and independent supplementary neuroblastoma mRNA expression datasets (Figure S1). Finally, univariate and multivariate regression analyses confirmed *ZRF1* to be independent prognostic factor of overall survival in neuroblastoma (Figure 1e,f).

**Figure 2:**
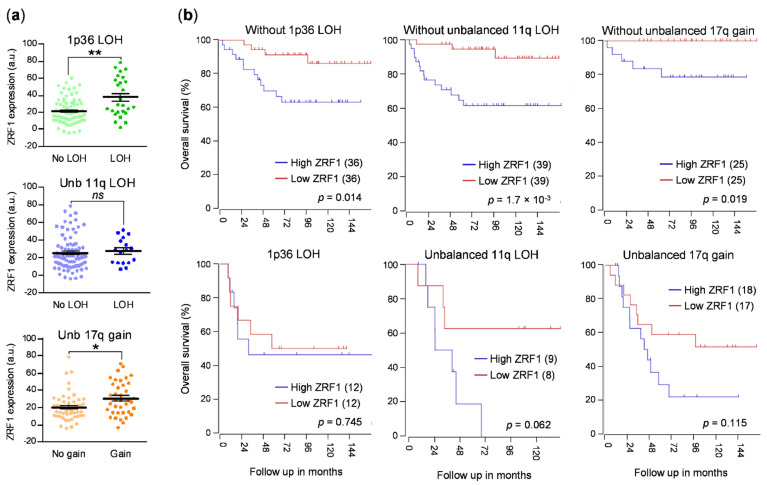
*ZRF1* levels correlate with low survival in the absence of genomic alterations associated with poor prognosis. (**a**) *ZRF1* mRNA levels in the presence or absence of the indicated segmental copy alterations (GSE3960, *n* = 101). (**b**) Kaplan–Meier survival plots comparing high and low *ZRF1* samples from the GSE3960 dataset, in the absence or presence of the different segmental copy alterations. *ns* means non-significant; * means *p* < 0.05; ** means *p* < 0.01.

Next, we analyzed the expression of *ZRF1* mRNA in patients with the most common segmental copy alterations associated with neuroblastoma prognosis, including 1p36 loss of heterozygosity (LOH), unbalanced 11q LOH, and unbalanced 17q gain [20,21]. *ZRF1* expression levels were found to be higher in patients with 1p36 loss and 17q gain (Figure 2a). A similar trend was also observed in patients with loss of 11q, although the difference was not statistically significant. Of note, the association between *ZRF1* expression and poor prognosis was maintained in tumors without these alterations (Figure 2b), further supporting the expression of *ZRF1* as an independent prognostic factor.

Analysis of *ZRF1* protein expression by Western blot on 24 tumor frozen samples from a cohort of 22 neuroblastoma patients was performed to confirm the mRNA results (Figure S2). Two pairs of samples corresponded to the same patients at different stages of the disease: early tumor resection or biopsy, and resection of metastatic lesions, respectively (Table S6). In most of the samples, the intensity of the *ZRF1* band was low or barely detectable, using SK-N-BE(2) cell line lysates as positive control. However, it is interesting to point out that the cases with higher expression of *ZRF1* were those corresponding to relapsed or metastatic neuroblastoma, thus supporting the fact that high *ZRF1* levels are present in the most aggressive neuroblastomas. Nevertheless, these results are preliminary and should be validated in a larger cohort of matched neuroblastoma samples.

Since most tumors contain heterogeneous cell populations, including malignant cells, immune cells, fibroblasts, and vascular cells, we proceed to confirm by immunohistochemistry that the *ZRF1* signal was from tumor cells. Figure S3 shows a representative image of a neuroblastoma tumor where the stromal component of the tumor shows a weak *ZRF1* immunoreactivity while tumor cells are highly positive.

In summary, *ZRF1* mRNA levels are associated with poor prognosis in neuroblastoma and may be used as an independent prognostic marker in the absence of MYCN amplification and other poor prognosis-related chromosomal aberrations.

### 3.2. ZRF1 Is Not Sufficient to Enhance Neuroblastoma Aggressiveness

**Figure 3:**
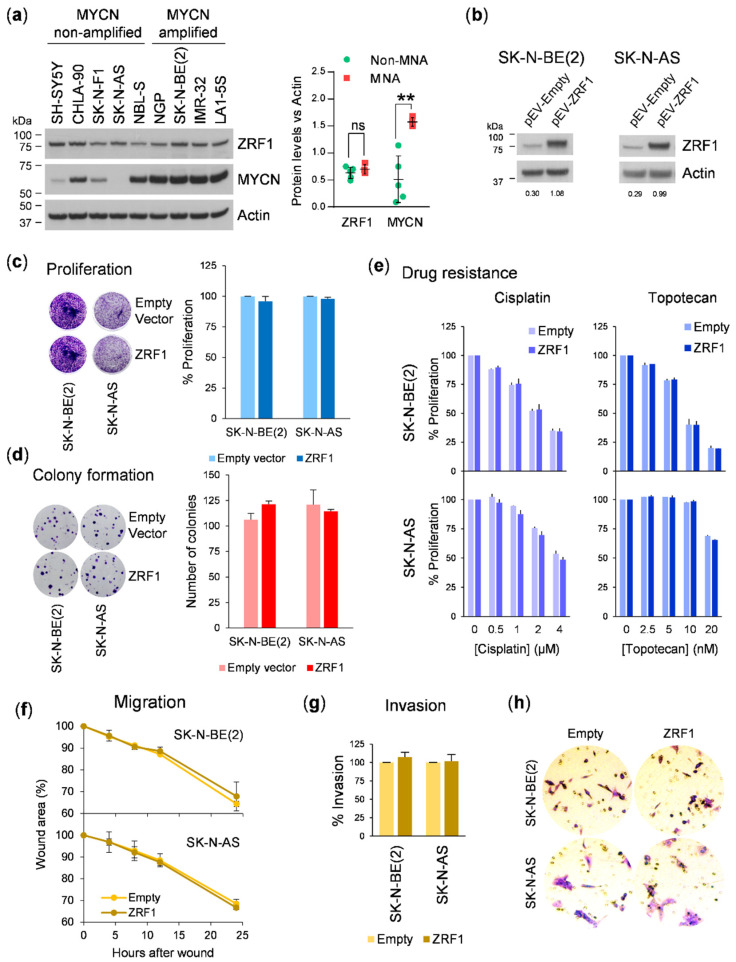
*ZRF1* overexpression does not enhance neuroblastoma cell line aggressiveness. (**a**) Left, protein expression analysis of *ZRF1* and MYCN by Western blot of a 9-neuroblastoma cell line panel. Right, densitometry quantification. (**b**) Western blot validation of *ZRF1* overexpression in SK-N-BE(2) and SK-N-AS cell lines at 96 h post-transduction. (**c**) Proliferation assay of neuroblastoma cell lines overexpressing *ZRF1* compared to empty-vector (pEV-empty)-transduced cells. (**d**) Colony formation assay of neuroblastoma cells overexpressing *ZRF1*. Graph represents the average of three independent experiments, *n* = 3 per condition. (**e**) Cisplatin and topotecan resistance assay of *ZRF1*-overexpressing cell lines. Cells were treated for 72 h at the indicated doses, and proliferation was assessed by crystal violet staining. (**f**) Wound-healing assay in neuroblastoma cells overexpressing *ZRF1*. Graphs represent percentage of the wound area at the indicated times, normalized to time = 0. (**g**) Invasion assay of *ZRF1* overexpressing cells through a collagen barrier for 16 h. Invasive cells were detected and quantified by crystal violet staining. (**h**) Representative microscopic pictures of crystal violet-stained invasive cells. *ns* means non-significant; ** means *p* < 0.01.

Given the role of *ZRF1* in the regulation of neuronal differentiation and the observed correlations in neuroblastoma samples, we studied the functional consequences of *ZRF1* overexpression in neuroblastoma cell lines. Protein expression analysis showed homogeneous levels of *ZRF1* among a panel of different neuroblastoma cell lines, regardless of MYCN amplification status (Figure 3a). To explore whether increasing the levels of *ZRF1* enhanced neuroblastoma aggressiveness, we transduced the SK-N-BE(2) and SK-N-AS neuroblastoma cell lines with a *ZRF1* lentiviral overexpression vector (Figure 3b), and then analyzed the effects of *ZRF1* on different oncogenic properties. Overexpression of *ZRF1* did not enhance proliferation (Figure 3c) or ability to form colonies when cells were plated at a low density (Figure 3d). Drug sensitivity assays were performed against cisplatin, an alkylating agent, and topotecan, an inhibitor of topoisomerase-I, which are two of the neuroblastoma standard-of-care chemotherapies. However, overexpression of *ZRF1* did not produce an increased resistance of neuroblastoma cells after 72 h of treatment (Figure 3e).

Owing to the lineage-conferring migratory capability of neuroblastoma cells, we next explored whether higher levels of *ZRF1* alters the migration or the invasion of cells in wound-healing and transwell assays, respectively. *ZRF1*-transduced cells closed the wound at the same pace as empty vector-infected cells (Figure 3f). Moreover, invasion through a collagen barrier was not affected by *ZRF1* overexpression in transwell assays (Figure 3g,h), thereby suggesting that *ZRF1* does not modulate the ability of neuroblastoma cells to migrate or invade.

**Figure 4:**
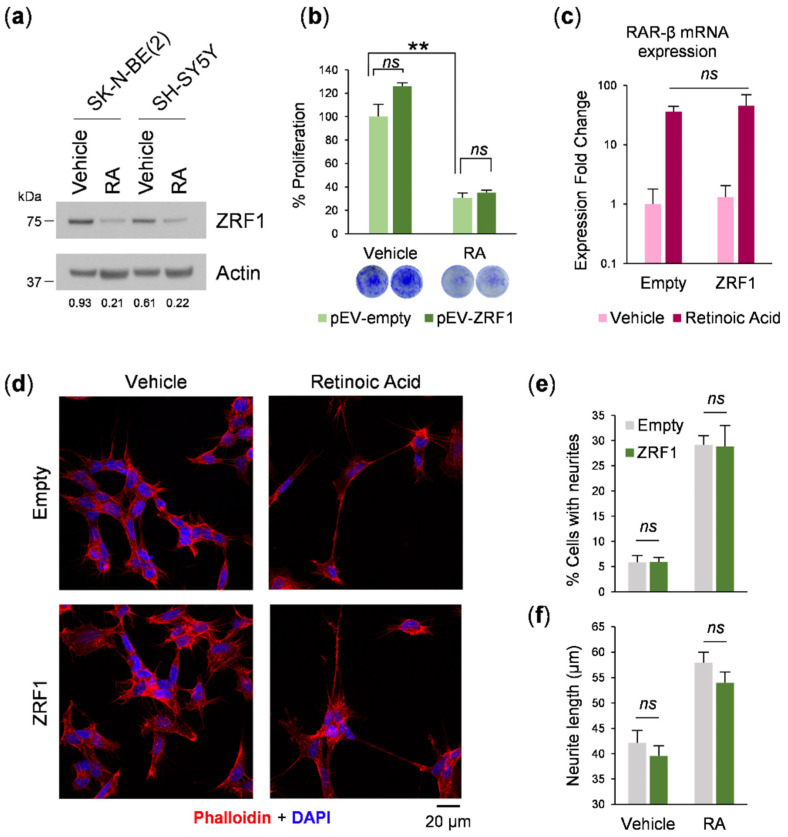
*ZRF1* overexpression does not attenuate retinoic acid-induced differentiation. (**a**) *ZRF1* levels after RA-induced differentiation measured by western blot. Actin-normalized densitometry quantification of *ZRF1* levels is shown beneath their respective Western blot panels (**b**) Cell proliferation assay of SK-N-BE(2) cells overexpressing *ZRF1* treated with RA and normalized versus empty vector-transduced cell treated with vehicle. (**c**) mRNA levels of the RA-induced differentiation reporter RAR-β, assessed by RT-qPCR in SK-N-BE(2) cells. (**d**) Confocal microscopy representative images of ZRF-overexpressing SK-N-BE(2) cells stained with phalloidin and DAPI. (**e**) Quantification of the number of cells with neurites (>30 µm prolongations) per field. (**f**) Average neurite length for each group. *ns* means non-significant; ** means *p* < 0.01.

13-*cis*-retinoic acid (hereafter referred to as RA) is a naturally occurring differentiating and therapeutic agent for the treatment of neuroblastoma minimal residual disease [6,22]. Because *ZRF1* has been previously demonstrated to be involved in maintaining neural progenitor stemness [8] and in altering retinoic acid induced differentiation [16], we analyzed the effect of modulating *ZRF1* in RA-differentiated neuroblastoma cells. After 5 days of RA treatment, *ZRF1* levels were found to be decreased 3–4 times in the RA-treated SH-SY5Y and SK-N-BE(2) neuroblastoma cell lines, as compared with vehicle-treated cells (Figure 4a). To evaluate the function of *ZRF1* in the process of RA-mediated differentiation, neuroblastoma cell lines overexpressing *ZRF1* were treated with the differentiating agent and the differentiation outcomes were analyzed. First, *ZRF1* overexpression did not rescue or attenuate the decrease in proliferation induced by RA-induced differentiation (Figure 4b); secondly, *ZRF1* overexpression did not alter the RA-induced upregulation of the RA receptor RAR-β(Figure 4c); finally, neither the percentage of cells with neurites (Figure 4d,e) nor the neurite length (Figure 4f) were modulated by overexpressing *ZRF1*.

These results indicate that sustained high levels of *ZRF1* are not enough to enhance oncogenic properties in neuroblastoma cells or impair RA-mediated neuroblastoma differentiation.

### 3.3. ZRF1 Knockdown Does Not Impair Neuroblastoma Proliferation and Reveals Inconsistencies between Different Gene Silencing Methodologies

**Figure 5:**
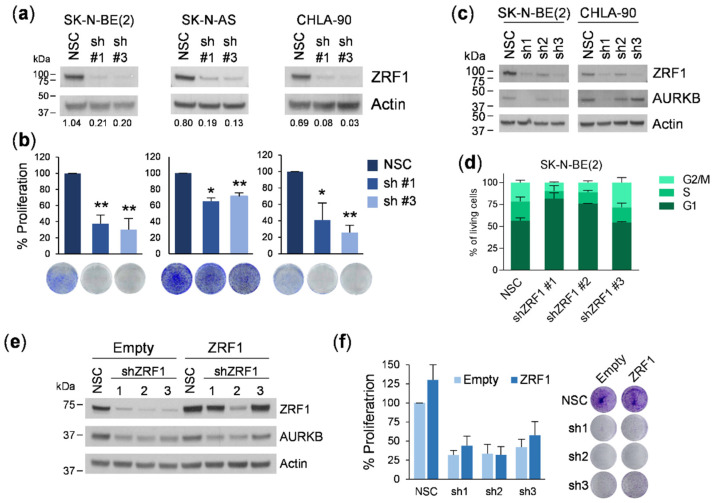
*ZRF1* shRNA silencing results in inconsistent and unspecific effects in neuroblastoma cells. (**a**) *ZRF1* expression levels in neuroblastoma cells transduced with two different shRNAs against *ZRF1*, and a non-silencing control (NSC) as negative control. (**b**) Cell proliferation in shZRF1-transduced neuroblastoma cell lines compared with those transduced with NSC, measured by crystal violet. (**c**) *ZRF1* and AURKB levels of sh*ZRF1* transduced neuroblastoma cell lines. (**d**) Cell cycle analysis of SK-N-BE(2) 72 h post-infection by FACS. (**e**) Western blot analysis of *ZRF1* and AURKB levels in SK-N- BE(2) cells overexpressing *ZRF1* insensitive to shZRF1, at 96h post-transduction. (**f**) Cell proliferation assay of SK-N-BE(2) cells overexpressing insensitive *ZRF1* transduced with 3 shRNAs against *ZRF1*. * means *p* < 0.05; ** means *p* < 0.01.

As *ZRF1* expression was noted in all the neuroblastoma cell lines tested, loss of function experiments represented a good strategy to fully dissect any relevant function of *ZRF1* in neuroblastoma cells. Thus, we knocked down *ZRF1* using lentiviral vectors. Two different shRNAs targeting the *ZRF1* coding region were transduced in six different neuroblastoma cell lines and showed a marked reduction in *ZRF1* levels compared to non-silencing control (NSC)-transduced cells (Figure 5a and Figure S4A). In all tested cell lines, shRNA-mediated depletion of *ZRF1* significantly reduced the proliferative capacity of neuroblastoma cells (Figure 5b and Figure S4B).

Transcriptomic analyses of shRNA-mediated *ZRF1* depleted cells showed the involvement of several genes related to the cell cycle (Figure S4C,D). When we validated the expression of some of those genes (i.e., AURKB), discrepancies among the molecular effects of the three different shRNAs were observed. For example, while one of the shRNAs (shRNA #1) completely abolished the expression of AURKB, the other two (shRNA #2 and #3) did not (Figure 5c). Cell cycle analyses in *ZRF1*-depleted cells also showed different profiles. The shRNAs #1 and #2 against *ZRF1* showed an increase in the percentage of cells in the G1 phase, whereas shRNA #3 showed a trend towards G2/M arrest (Figure 5d). To discard potential shRNA off-target effects, we performed rescue experiments by overexpressing a *ZRF1* variant with silent mutations in the shRNA target sites (Figure S5). This new mutant *ZRF1* was completely insensitive to shRNAs #1 and #3, and partially sensitive to shRNA #2 (Figure 5e). When the phenotypic effects were analyzed, the ectopic expression of *ZRF1* did not rescue the reduction in proliferation caused by the transduction of the three different shRNAs (Figure 5f). Thus, we concluded that the phenotypic consequences induced by the different shRNAs were not attributable to *ZRF1* depletion.

**Figure 6:**
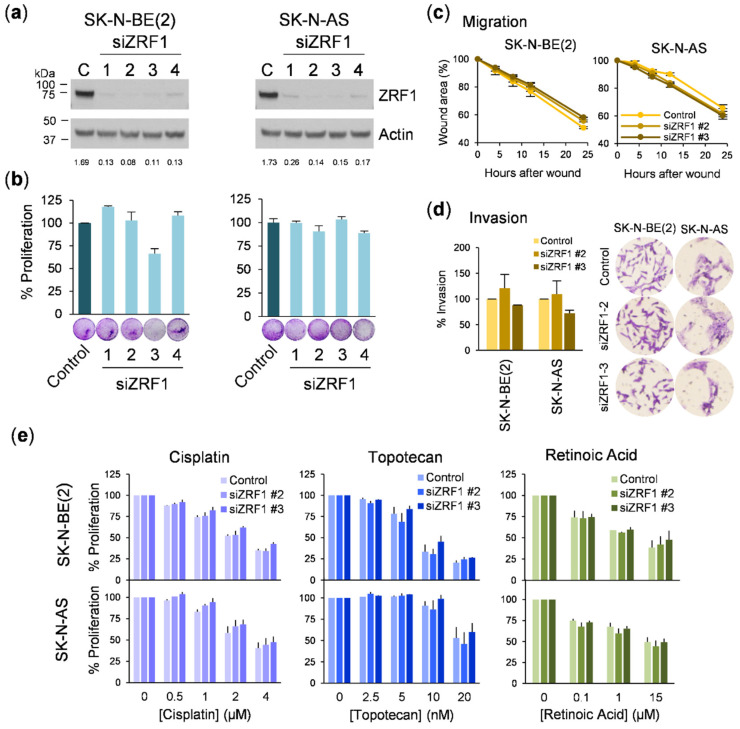
*ZRF1* is dispensable for neuroblastoma growth, migration, and drug resistance. (**a**) *ZRF1* levels in neuroblastoma cells transfected with siRNA control and 4 different si*ZRF1*. Actin-normalized densitometry quantification of *ZRF1* levels is shown beneath their respective *ZRF1* knockdown validation Western blot panels (**b**) Cell viability assay in neuroblastoma cells transfected comparing siControl vs si*ZRF1* at 96h post-transfection. (**c**) Wound-healing assay in si*ZRF1* transfected neuroblastoma cells at 72 h post-transfection. (**d**) Invasion assay through a collagen barrier of si*ZRF1* transfected cells at 72 h post-transfection. Left, crystal violet quantification of invaded cells. Right, representative images of the invasion assay. (**e**) Cisplatin (left), topotecan (middle), and retinoic acid (right) resistance assay of si*ZRF1*-transfected cell lines. Cells were treated after 72 h of transfection at the indicated doses for 72 h more and proliferation was assessed by crystal violet staining.

To exclude the possibility that some of the *ZRF1* knockdown effects were masked by the shRNA off-targets, we repeated the experiments with small interfering RNA (siRNA). Up to four different siRNA sequences were transfected into neuroblastoma cells. All the sequences reduced the *ZRF1* level by more than 85% (Figure 6a). When the phenotypic consequences of siRNA-mediated *ZRF1* depletion were analyzed, no differences were found in cell proliferation (Figure 6b), wound healing (Figure 6c), invasion through collagen (Figure 6d), or resistance to neuroblastoma therapies such as chemotherapeutics or RA (Figure 6e).

In summary, our results indicate that *ZRF1* has a prognostic value, but is not functionally relevant in neuroblastoma cells. 

## 4. Discussion

Neuroblastoma is thought to originate from cells of the neural crest that are transformed during differentiation and migration toward tissues of the sympathoadrenergic lineage. The occurrence of a transformative event during tissue differentiation dictates the aggressiveness of the tumor. In general, patients with poor prognosis have histologically undifferentiated tumors, whereas those with better prognosis have tumors with histological evidence of cellular differentiation [23]. This differentiation program is tightly regulated by a complex set of signals, including external signaling, activation of specific transcriptional programs, and/or epigenetic events (reviewed in [24]). Experimental results in transgenic mouse models have identified activating ALK mutations and MYCN overexpression as the main oncogenic drivers of neuroblastoma [25,26]. These molecular alterations often converge on mechanisms that block differentiation and confer sustained proliferation capabilities. In particular, genes associated with the maintenance of embryonic and adult stem cells, such as the components of PRC1 or PRC2, have been linked to the initiation and progression of neuroblastoma [27]. For example, BMI1, a core component of the PRC1 complex, has been shown to cooperate in MYCN-driven neuroblastomas by inhibiting cell death and differentiation [28,29,30]. Focusing on the role of PRC1 in neuronal differentiation, Aloia et al. identified *ZRF1* as a transcriptional regulator of neural fates in embryonic stem cells [9]. Furthermore, *ZRF1* expression has been associated with poor outcomes in other tumors, such as breast [14,31] or gastric cancer [15], thus suggesting an oncogenic role in cancer. Thus, we sought to determine whether *ZRF1* plays a functional role in aggressive neuroblastomas. Data mining of multiple neuroblastoma gene expression datasets confirmed that *ZRF1* mRNA expression was elevated in subsets of patients with the most common genetic alterations associated with poor outcomes, thereby supporting our initial hypothesis, although this correlation could not be confirmed at the protein level in neuroblastoma patient samples.

Nevertheless, our functional data do not support a relevant contribution of *ZRF1* in the oncogenesis of neuroblastoma. Ectopic expression of *ZRF1* did not offer proliferation, colony formation, or migratory or invasive advantages to neuroblastoma cells. In acute myeloid leukemia, *ZRF1* is a regulator of RA-induced differentiation [16], and because of the relevance of retinoids in neuroblastoma therapy [22], we investigated whether *ZRF1* interferes with the response of neuroblastoma cells to RA. When neuroblastoma cells were exposed to 13-*cis*-RA, a clear reduction in the *ZRF1* level was observed, concomitant with an expected reduction in cell proliferation and morphological changes such as neurite outgrowth. However, maintaining high *ZRF1* expression ectopically was not sufficient to reverse the RA effect. These results suggest that the downregulation of *ZRF1* is a consequence of the RA-induced differentiation process.

Although *ZRF1* is not sufficient to provide oncogenic advantages, it could still be necessary to maintain the undifferentiated and highly proliferative state of neuroblastoma cells. Previous reports demonstrated that depletion of *ZRF1* resulted in a reduction of cell proliferation and the induction of apoptosis in gastric [15] or breast cancer models [14]. Conversely, Kaymak et al. also showed that the reduction in cell proliferation mediated by *ZRF1* depletion was accompanied by an increase in the migration and invasion properties of breast cancer cells [31]. Our first set of experiments silencing *ZRF1* using two different shRNAs resulted in a marked reduction in cell proliferation in six neuroblastoma cell lines. However, these effects were not rescued by overexpressing the shRNA-insensitive form of *ZRF1*, thereby indicating that the depletion of *ZRF1* was not the causal factor for the reduction in cell proliferation. Consistent with this previous finding, siRNA-mediated depletion of *ZRF1* did not alter the proliferative, migratory, or invasive capacities or the sensitivity to RA in neuroblastoma cells, thus confirming that *ZRF1* is dispensable for the progression of this type of tumor. It is important to highlight that inconsistencies between different gene silencing methods could have misled the conclusion of this study. However, our use of independent silencing tools and rescue experiments with target protein ectopic expression leaves no room for doubt.

Imamura et al. demonstrated that while the effects of *ZRF1* on cell proliferation were p53-dependent, those related to migration and invasion were p53-independent [15]. In our work, we covered this aspect by using cell lines with non-functional (CHLA-90, SK-N-BE(2), and SK-N-AS) and functional p53 (SH-SY5Y and IMR-32), and we did not find any differential response in the gain-of-function or loss-of-function experiments.

The paradoxical discrepancy between the strong correlation between *ZRF1* expression and poor patient outcome, and its dispensable function in tumor cells could be due to one or more of the following reasons: (i) one key characteristic of cancer is uncontrolled transcription. Thus, many genes are likely to be differentially expressed incidentally, rather than reflecting a gene that is driving a biologically significant outcome [32]; (ii) the *ZRF1*-PRC1 axis may regulate different sets of genes in a lineage-dependent manner; (iii) molecular alterations present in neuroblastoma (i.e., mutation burden, chromosomal copy number variations, etc.) deactivate the physiological regulation of *ZRF1*-PRC1 on cell proliferation/differentiation programs and become *ZRF1* independent; and (iv) the ambivalent molecular function of *ZRF1* in neuroblastoma cells may be inclined to a non-essential chaperone role. This hypothesis is supported by our subcellular fractionation analysis in different neuroblastoma cell lines, where *ZRF1* was found to be predominantly enriched in the cytosolic fraction (Figure S6). 

In summary, our data suggest the potential use of *ZRF1* expression as an independent prognostic factor, particularly in cases without any other associated molecular prognostic factors. However, *ZRF1* does not seem to be a promising target candidate for differentiation therapy for neuroblastoma.

## 5. Conclusions

*ZRF1* was found to be an independent prognostic factor of survival in neuroblastoma. However, this correlation cannot be explained by the molecular role of *ZRF1* by itself, and it could be the reflection of an underlying molecular mechanism promoting neuroblastoma aggressiveness. Nevertheless, our functional studies highlight the need of proper verification of shRNA-mediated knockdown experiments through consistent validation with different gene silencing technologies and rescue experiments.

## Data Availability

Publicly available datasets were analyzed in this study. These data can be found at http://r2.amc.nl under the accession numbers GSE45547, GSE3960, GSE62564.

## Supplementary Materials

The following are available online at https://www.mdpi.com/article/10.3390/cancers13194845/s1, Figure S1: *ZRF1* is associated to poor prognosis in neuroblastoma. Figure S2: *ZRF1* protein levels Western blot analysis in patient tumor samples. Figure S3: *ZRF1* protein is detected by immunohistochemistry (IHC) in the tumor cells of a neuroblastoma formalin-fixed paraffin-embedded human sample. Figure S4: Functional and molecular consequences of shRNA-mediated depletion of *ZRF1* in neuroblastoma cells. Figure S5: Design of a triple shRNA insensitive *ZRF1* overexpression construct. Figure S6: Subcellular localization analysis of *ZRF1* by Western blot in a panel of neuroblastoma cell lines. Figure S7. Original Western Blot image of Figure 3a,b, Figure 4a, Figure 5a,c,e, Figure 6a, Figure S2a and Figure S4. Table S1: Antibodies used for Western blot analyses. Table S2: Oligonucleotides used for silent *ZRF1* mutagenesis and RARB RT-qPCR. Table S3: *ZRF1* targeting sequences. Table S4: Cut-off value for *ZRF1* levels analysis, based on the ability for overall survival prediction (using GSE62564, *n* = 498). Table S5: *ZRF1* mRNA expression correlations with clinical variables in neuroblastoma using Fisher’s test (GSE62564, *n* = 498). Table S6: Neuroblastoma tumor sample clinical data.
