# Supplementary material for: Neuronal Differentiation-Related Epigenetic Regulator ZRF1 Has Independent Prognostic Value in Neuroblastoma but Is Functionally Dispensable In Vitro
Source: Cancers (Basel). 2021 Sep 28;13(19):4845. doi: 10.3390/cancers13194845 (PMC8508520; doi:10.3390/cancers13194845)
Supplement: Supplementary file 1 [file cancers-13-04845-s001.zip › cancers-1376300-supplementary.pdf]

cancers

MDPI

Article

Neuronal Differentiation-Related Epigenetic Regulator *ZRF1* Has Independent Prognostic Value in Neuroblastoma but Is Functionally Dispensable In Vitro

Carlos Jiménez [1], Roberta Antonelli [1], Marc Masanas [1], Aroa Soriano [1], Laura Devis-Jauregui [2,3], Jessica Camacho [4], Ainara Magdaleno [1], Gabriela Guillén [1,5], Raquel Hladun [1,6], Luz Jubierre [1], Josep Roma [1], David Llobet-Navas [2,3], Josep Sánchez de Toledo [1,7], Lucas Moreno [1,6], Soledad Gallego [1,6] and Miguel F. Segura [1,*]

Supplementary

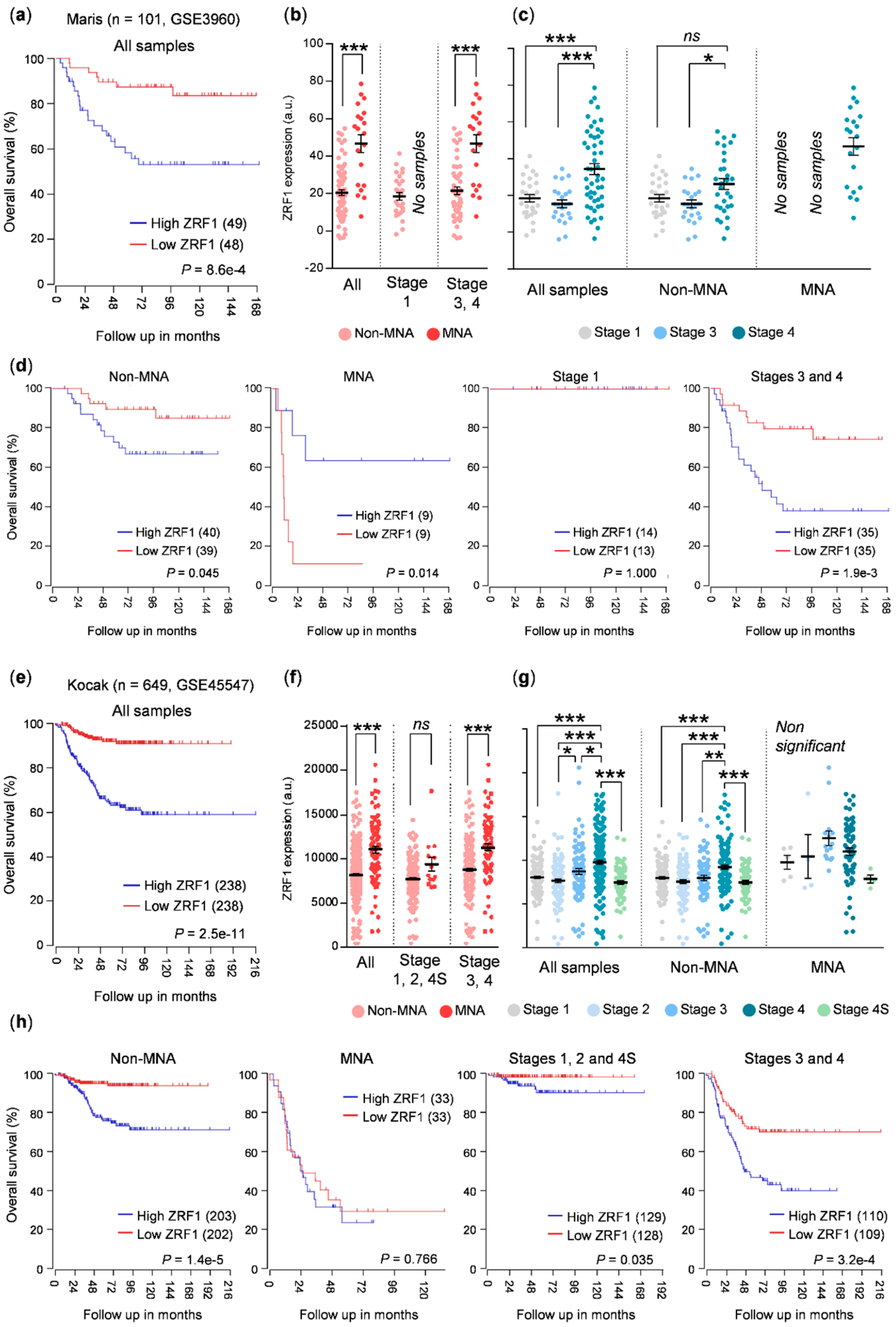

Citation: Jiménez, C.; Antonelli, R.; Masanas, M.; Soriano, A.; Devis-Jauregui, L.; Camacho, J.; Magdaleno, A.; Guillén, G.; Hladun, R.; Jubierre, L.; et al. Neuronal Differentiation-Related Epigenetic Regulator *ZRF1* has Independent Prognostic Value in Neuroblastoma but is Functionally Dispensable In Vitro. *Cancers* **2021**, *13*, 4845. https://doi.org/10.3390/cancers13194845

Academic Editors: Luis Franco and Rakesh K. Singh

Received: 26 August 2021
Accepted: 25 September 2021
Published: 28 September 2021

Publisher's Note: MDPI stays neutral with regard to jurisdictional claims in published maps and institutional affiliations.

Copyright: © 2021 by the authors. Licensee MDPI, Basel, Switzerland. This article is an open access article distributed under the terms and conditions of the Creative Commons Attribution (CC BY) license (https://creativecommons.org/licenses/by/4.0/).

Figure S1. ZRF1 is associated to poor prognosis in neuroblastoma. (**a**) Kaplan-Meier survival plot of a cohort of 101 patients (GSE3960) split in high (above median) and low (below median) ZRF1 mRNA expression. (**b**) ZRF1 mRNA levels in MYCN-ampli-fied tumors (MNA, dark red) vs non-MYCN amplified tumors (non-MNA, light red) according to the indicated disease stages. (**c**) ZRF1 mRNA levels according to disease stage in the whole cohort (left) or considering patients with non-MNA (middle) or with MNA tumors (right). (**d**) Kaplan-Meier survival plots comparing samples with high (above median) and low (below median) ZRF1 levels in the indicated groups of patients. (**e**) Kaplan-Meier survival plot of a cohort of 649 patients (GSE45547) split in high (above median) and low (below median) ZRF1 mRNA expression. (**f**) ZRF1 mRNA levels in MYCN-amplified tumors (MNA, dark red) vs non-MYCN amplified tumors (non-MNA, light red) according to the indicated disease stages. (**g**) ZRF1 mRNA levels according to disease stage in the whole cohort (left) or considering patients with non-MNA (middle) or with MNA tumors (right). (**h**) Kaplan-Meier survival plots comparing samples with high (above median) and low (below median) ZRF1 levels in the indicated groups of patients. *ns* means 'non-significant'; * means $P < 0.05$; ** means $P < 0.01$; *** means $P < 0.001$.

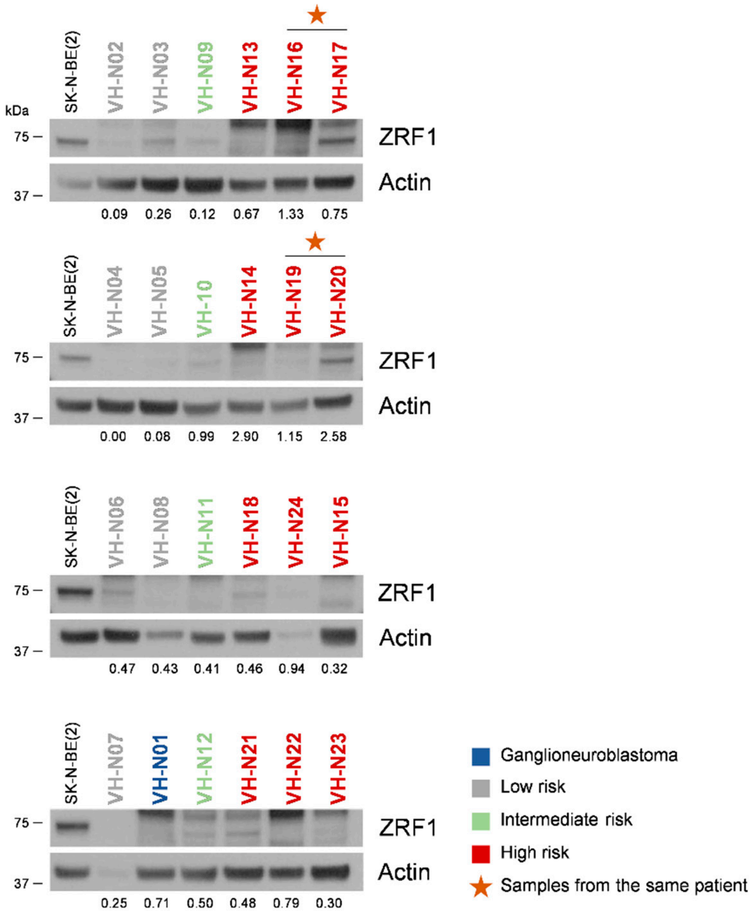

Figure S2. ZRF1 protein levels western blot analysis in patient tumor samples. Protein homogenates from a cohort of 22 neuroblas-toma patients were analyzed by western blot. Actin was used as loading control. Actin-normalized ZRF1 relative levels are shown underneath, expressed as Fold Change vs SK-N-BE(2) cell line. Stars indicate samples of the same patient.

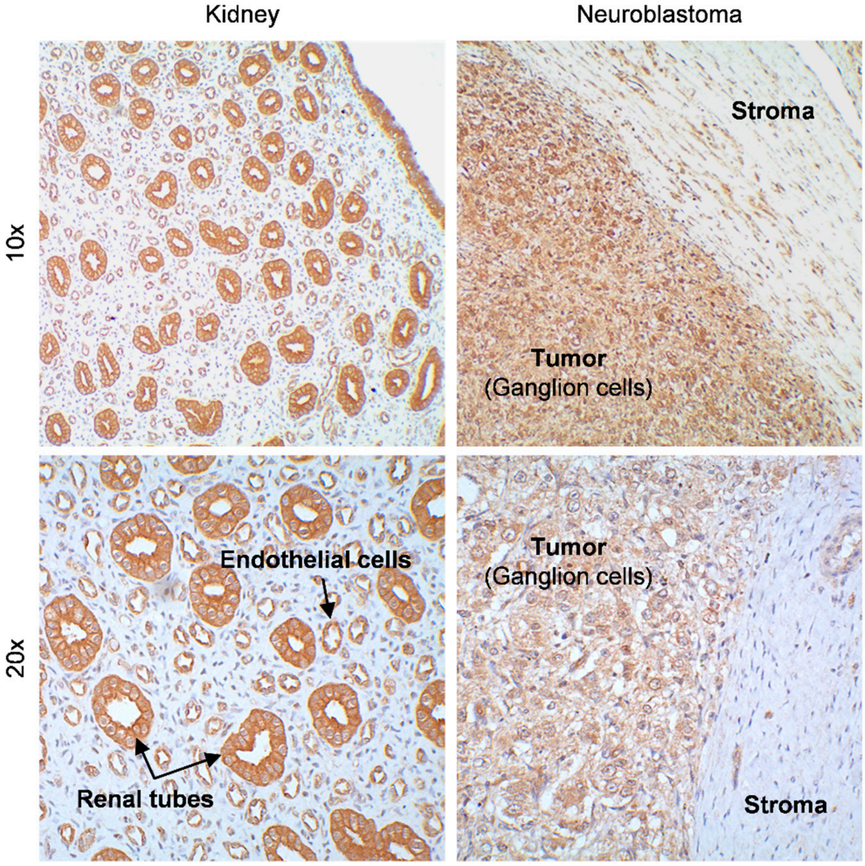

Figure S3. ZRF1 protein is detected by immunohistochemistry (IHC) in the tumor cells of a neuroblastoma formalin-fixed paraffin-embedded human sample. A kidney sample was used as control for specific staining (positive for renal tubules and endothelial cells, negative for fibroblasts).

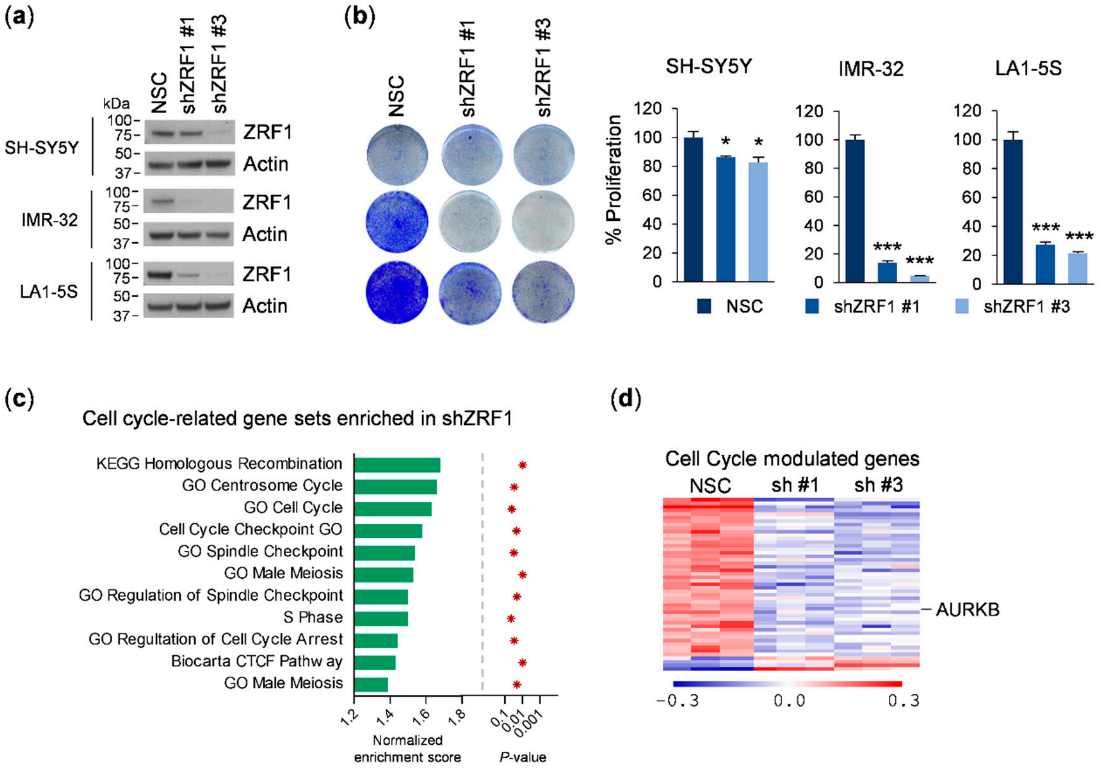

Figure S4. Functional and molecular consequences of shRNA-mediated depletion of ZRF1 in neuroblastoma cells. (a) ZRF1 protein levels upon shZRF1 #1 and #3 transduction in the indicated neuroblastoma cell lines. (b) Cell proliferation assay in neuroblastoma cell lines infected with NSC and shZRF1 for 96h. (c) Gene expression microarray data of SK-N-BE(2) cells transduced with NSC or shZRF1 #1 and #3, at 72h post-infection. Differential gene expression was analyzed by Gene Set Enrichment Analysis (GSEA). Normalized enrichment score and *P*-value of cell cycle-related gene sets from KEGG, GO, Biocarta and MSigDB Hallmarks are shown. (**d**) Heatmap showing expression levels of genes corresponding to cell cycle-related gene sets, including AURKB, in shZRF1 transduced SK-N-BE(2) cells. * means $P < 0.05$; *** means $P < 0.001$.

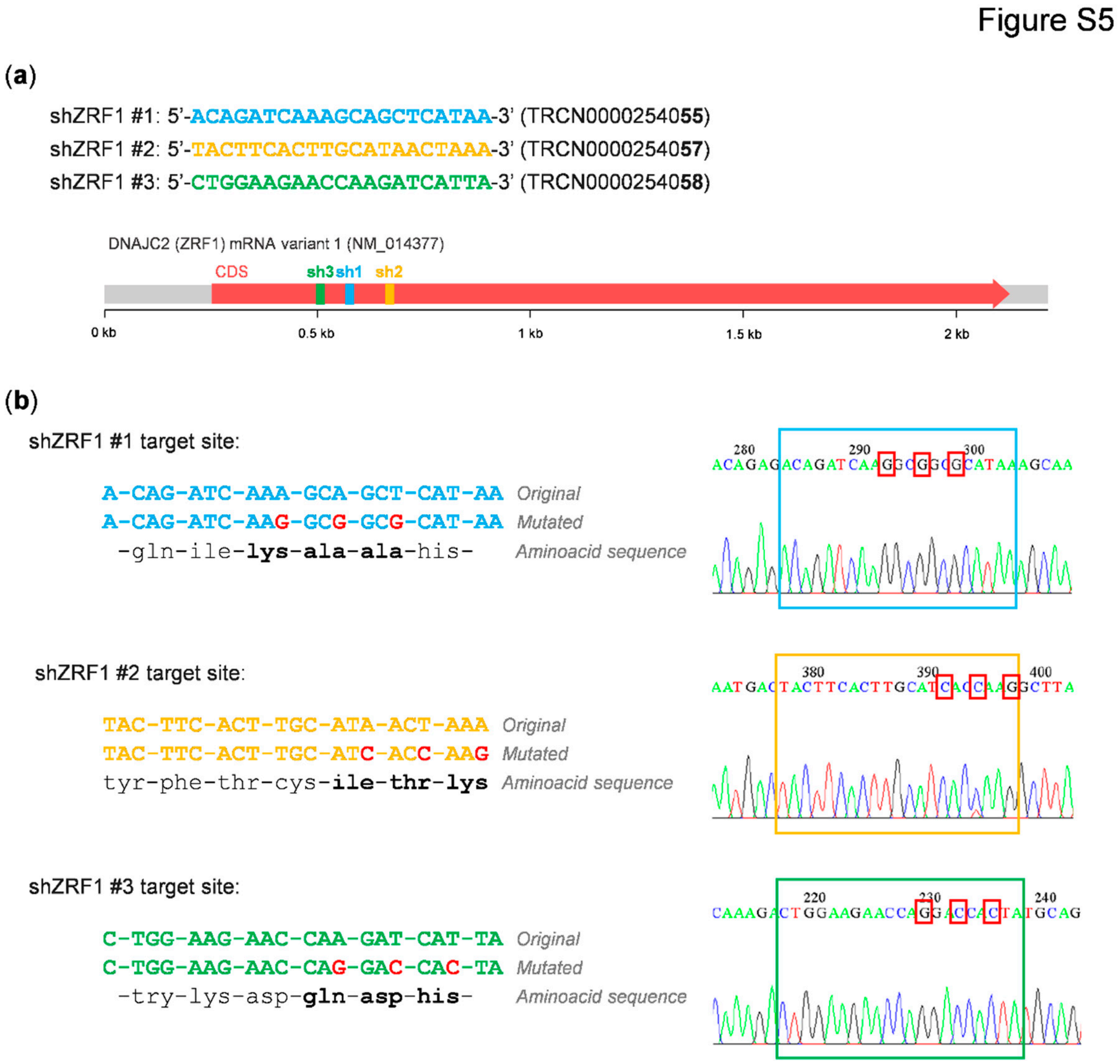

Figure S5. Design of a triple shRNA insensitive ZRF1 overexpression construct. (**a**) Target sequences of the three shRNAs used in this study and their position in ZRF1 (*DNAJC2*) mRNA. (**b**) Left, design of three silent mutations on each target sequence, generated by site-directed mutagenesis. Right, Sanger electropherogram validating the correct incorporation of these mutations into pCAG-ZRF1 cDNA vector.

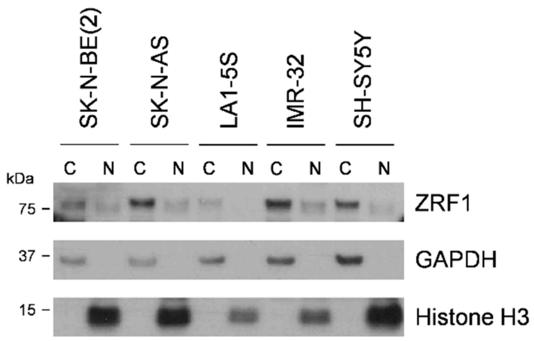

Figure S6. Subcellular localization analysis of ZRF1 by western blot in a panel of neuroblastoma cell lines. Nuclei were isolated from cytoplasmic (C) fraction by lysing cells in a hypotonic buffer and subjected to 50 strokes with a Potter Elvehjem homogenizer. Nuclear fraction (N) was extracted by pelleting and lysing the nuclei with RIPA buffer. GAPDH and histone H3 were used as cytoplasmic and nuclear markers, respectively.

Figure 3A

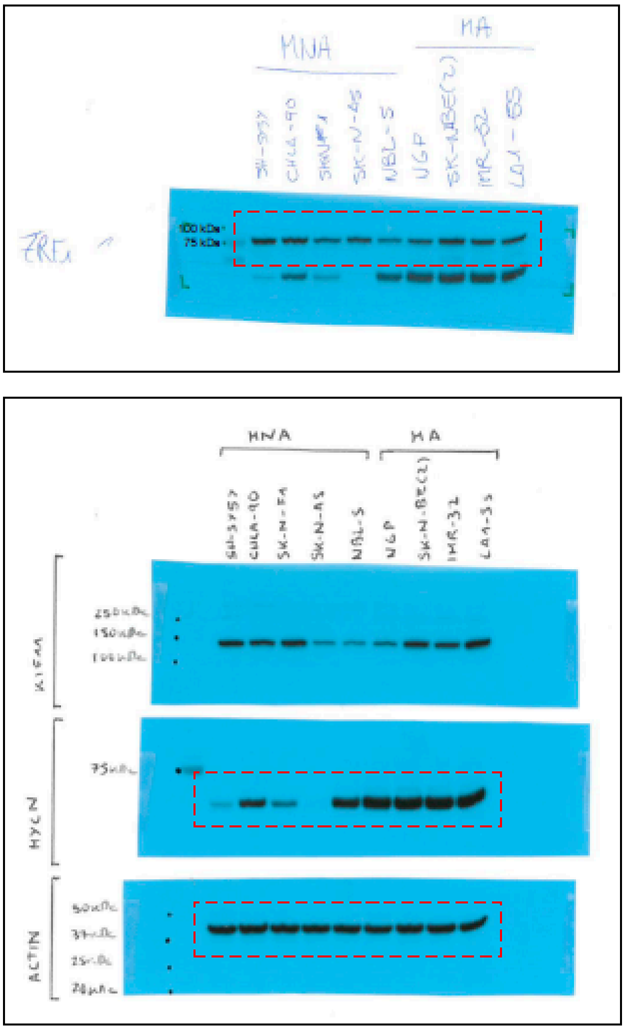

Figure 3B

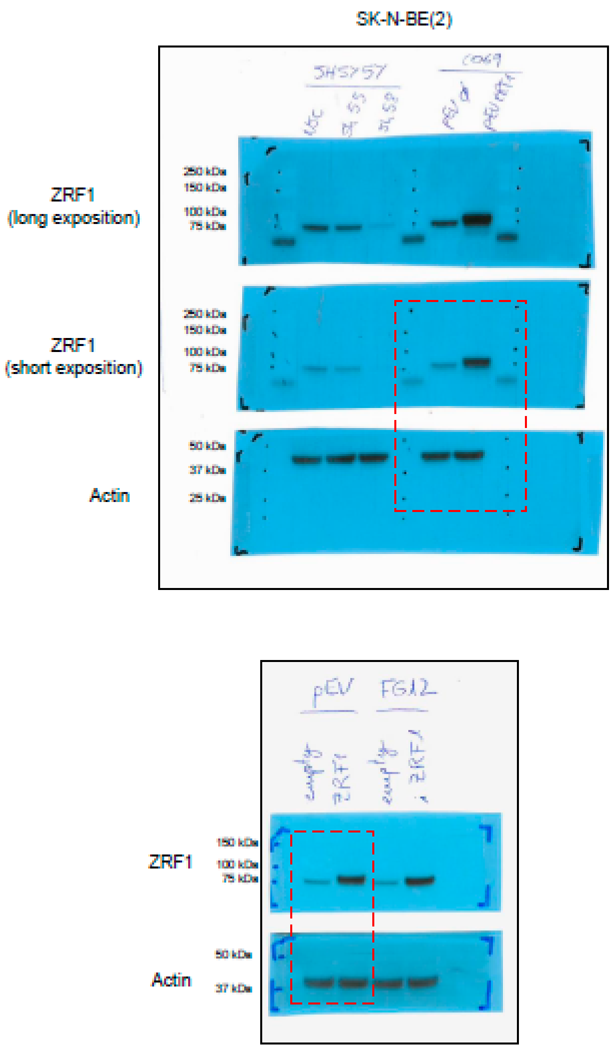
SK-N-BE(2)
ZRF1
(long exposition)
ZRF1
(short exposition)
Actin
250 kDa
150 kDa
100 kDa
75 kDa
50 kDa
37 kDa
25 kDa
pEV
FG12
empty
ZRF1
ZRF1
Actin

Figure 4A

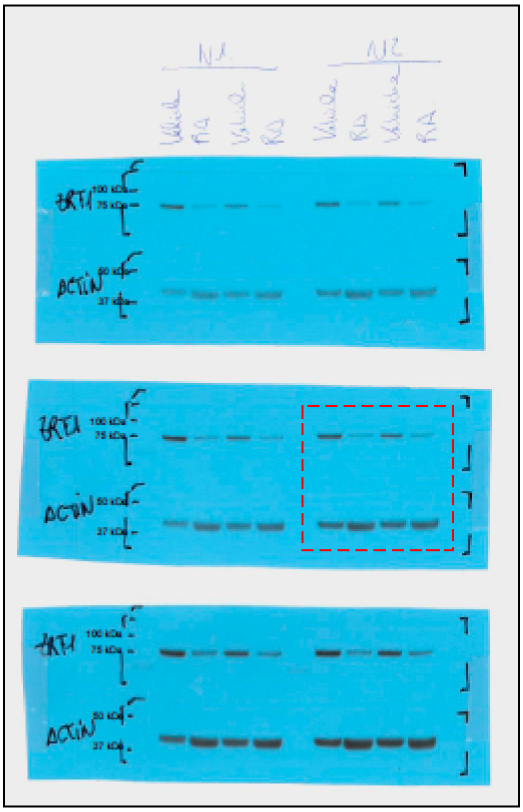

Figure 5A

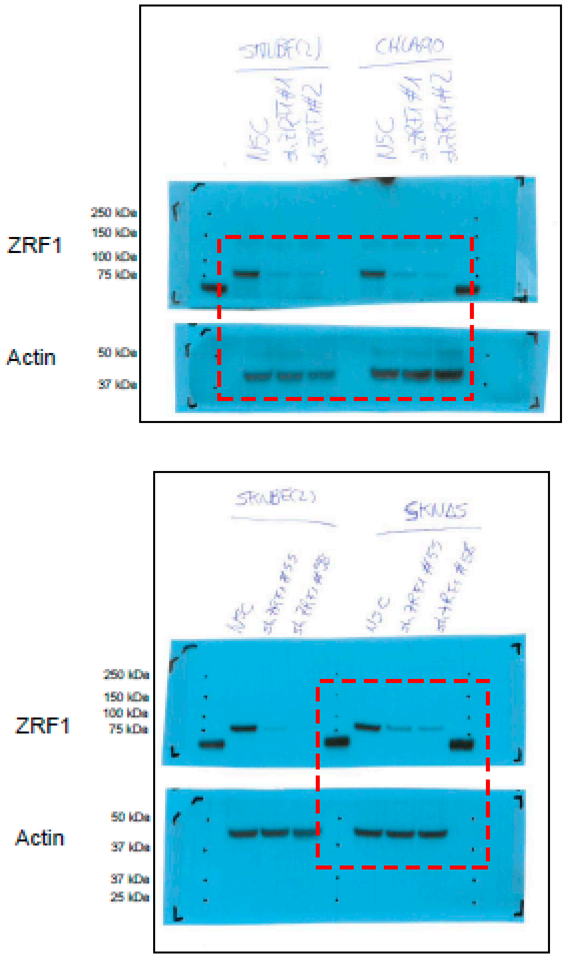
ZRF1
250 kDa
150 kDa
100 kDa
75 kDa
Actin
50 kDa
37 kDa
ZRF1
250 kDa
150 kDa
100 kDa
75 kDa
Actin
50 kDa
37 kDa
37 kDa
25 kDa

Figure 5C

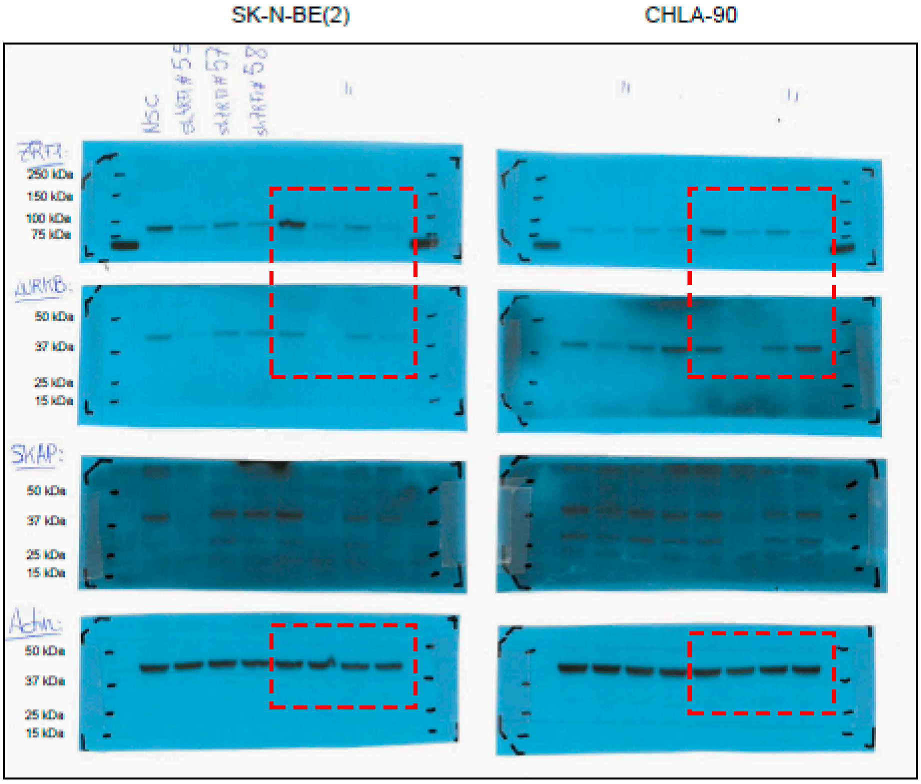
SK-N-BE(2)
CHLA-90
250 kDa
150 kDa
100 kDa
75 kDa
50 kDa
37 kDa
25 kDa
15 kDa

Figure 5E

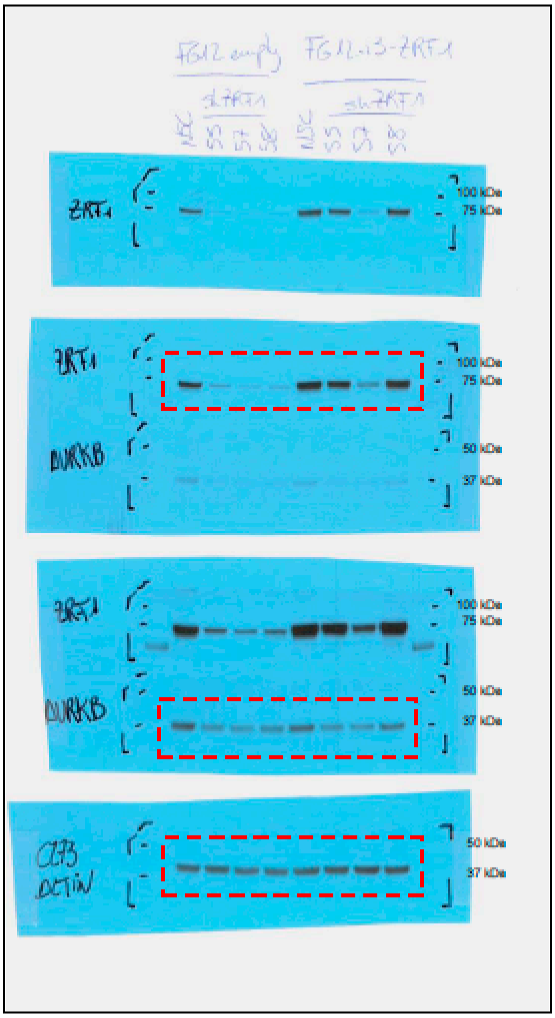
100 kDa
75 kDa
100 kDa
75 kDa
50 kDa
37 kDa
100 kDa
75 kDa
50 kDa
37 kDa
50 kDa
37 kDa

Figure 6A

SK-N-BE(2)

SK-N-AS

SK-N-BE(2)

SK-N-AS

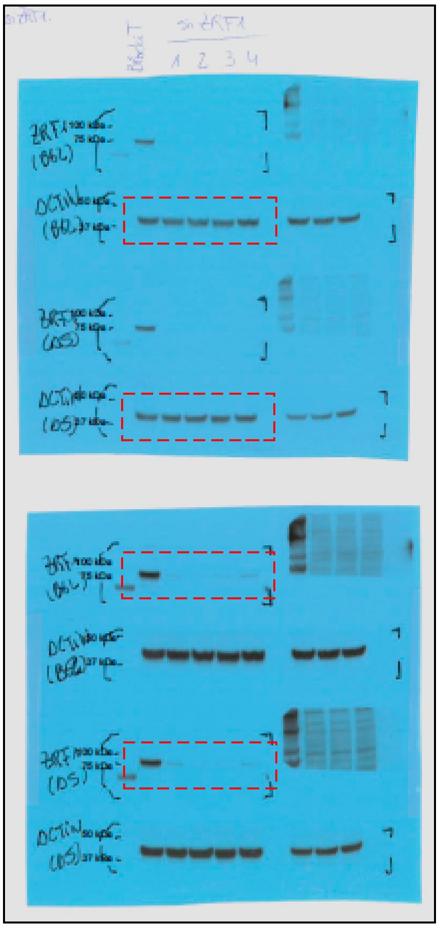

Figure S2A

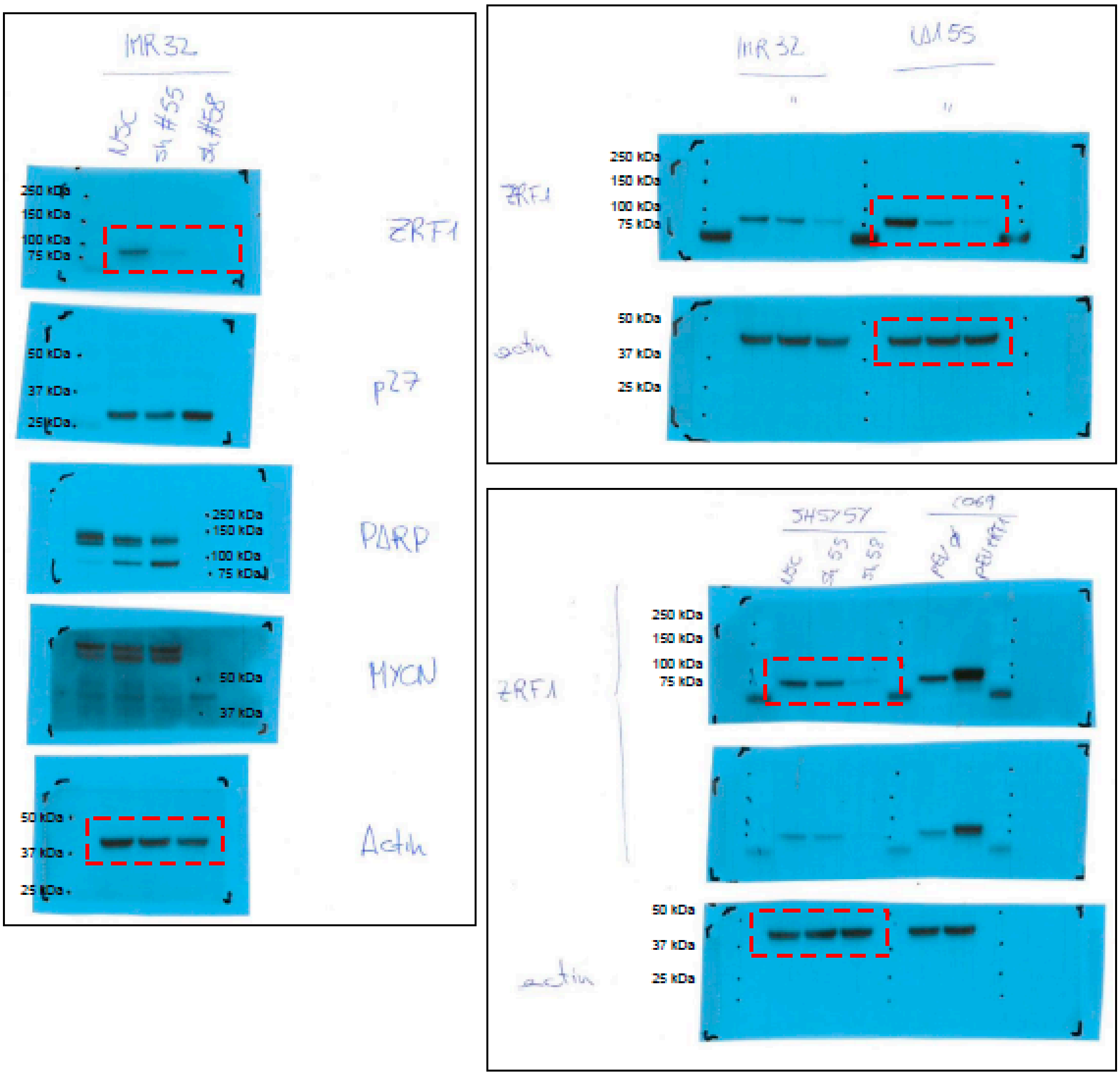
IMR32
250 kDa
150 kDa
100 kDa
75 kDa
ZRF1
50 kDa
37 kDa
25 kDa
p27
PARP
MYCN
Actin
actin

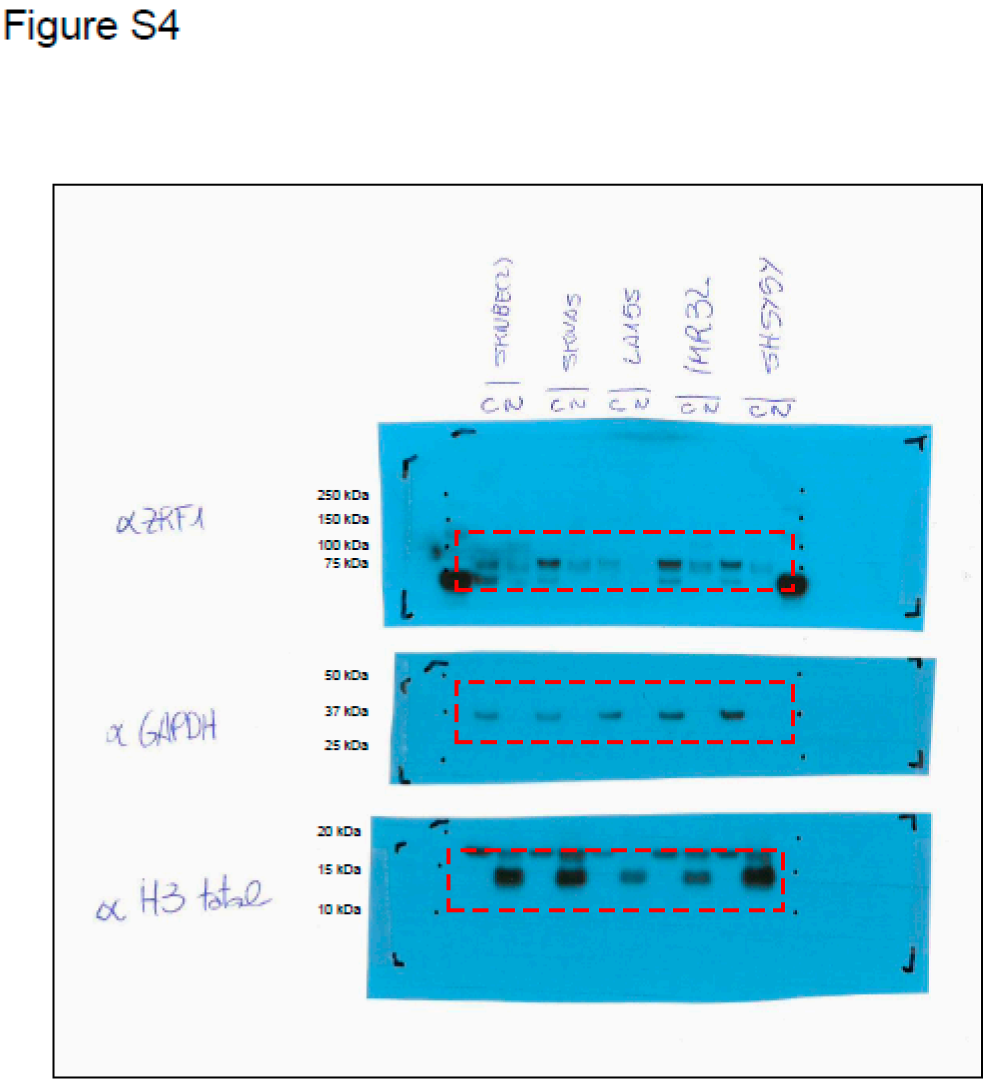

Figure S7. Original Western Blot image of Figure 3A, B, Future 4A, Figure 5A,C,E, Figure 6A, Figure S2A and Figure S4.

Table S1. Antibodies used for western blot analyses.

Protein	Origin	Dilution	Company	Reference
		Primary antibodies		
ZRF1	Rabbit	1:1000 in 5% milk	Provided by Luciano DiCroce	
MYCN	Mouse	1:2000 in 5% BSA	Santa Cruz Biotechnology	sc-53993
AURKB	Mouse	1:1000 in 5% BSA	BD Biosciences	#611083
β-actin	Mouse	1:10000 in 5% BSA	Santa Cruz Biotechnology	sc-47778 HRP
		Secondary antibodies		
α-rabbit IgG-peroxidase		1:10000	Sigma-Aldrich	#A0545
α-mouse IgG-peroxidase		1:10000	Sigma-Aldrich	#A9044

BSA: Bovine Serum Albumin; HRP: Horseradich Peroxidase. Headquarters: Santa Cruz Biotechnology, Dallas, TX, USA; BD Biosciences, San Jose, CA, USA; Sigma-Aldrich, St. Louis, MO, USA.

Table S2. Oligonucleotides used for silent ZRF1 mutagenesis and *RARB* RT-qPCR.

Name	Sequence
Primers	
ZRF1mut1_Fw	GCTACACAGAGACAGATCAAGGCGGCGCATAAAGCAATGG

ZRF1mut1_Rv	CCATTGCTTTATGCGCCGCCTTGATCTGTCTCTGTGTAGC
ZRF1mut2_Fw	CTTGCATCACCAAGGCTTATGAAATGTTATCTGATCCAG
ZRF1mut2_Rv	CATAAGCCTTGGTGATGCAAGTGAAGTAGTCATTATCTCC
ZRF1mut3_Fw	CCCAAAGACTGGAAGAACCAGGACCACTATGCAGTTCTTGG
ZRF1mut3_Rv	CCAAGAACTGCATAGTGGTCCTGGTTCTTCCAGTCTTTGGG
ZRF1mut_seq	ATGCTGCTTCTGCCAAGC
RARB_Fw	TCCGAAAAGCTCACCAGGAAA
RARB_Rv	GGCCAGTTCACTGAATTTGTCC
L27_Fw	AGCTGTCATCGTGAAGAA
L27_Rv	CTTGGCGATCTTCTTCTTGCC

Table S3. ZRF1 targeting sequences.

shRNA	
shZRF1 #1 (TRCN0000254055)	ACAGATCAAAGCAGCTCATAA
shZRF1 #2 (TRCN0000254057)	TACTTCACTTGCATAACTAAA
shZRF1 #3 (TRCN0000254058)	CTGGAAGAACCAAGATCATTA
siRNA	
siZRF1 #1	GAUCAAAGCAGCUCAUAAA
siZRF1 #2	GAACUUGUCGAGAUGGUAA
siZRF1 #3	CGACAAGCAUCUAAGAACA
siZRF1 #4	GUUAUCUGAUCCAGUGAAA

Table S4. Cut-off value for ZRF1 levels analysis, based on the ability for overall survival prediction (using GSE62564, *n* = 498).

Cut-off Point	Value	Sensibility/ Specificity
Median (d = 0.368)	20.5625	(0.790;0.578)
AUC OS (Youden Index d = 0.4450)	24.2395	(0.610; 0.835)
**OS ROC curve**: AUC = 0.765; *P*-value < 0.001		
AUC: area under the curve; OS: Overall Survival		

Table S5. ZRF1 mRNA expression correlations with clinical variables in neuroblastoma using Fisher's test (GSE62564, n = 498).

Variable	Σ	ZRF1		P value
		Low	High	
All patients	498	369 (74.1%)	129 (25.9%)	
**Sex**				
M	287	211 (73.5%)	76 (26.5%)	0.757
F	211	158 (74.9%)	53 (25.1%)	
**Age**				
<18 months	300	264 (88.0%)	36 (12.0%)	0.000
≥18 months	198	105 (53.0%)	93 (47.0%)	
**INSS Stage**				
1	121	116 (95.9%)	5 (4.1%)	0.000
2	78	68 (87.2%)	10 (12.8%)	
3	63	41 (65.1%)	22 (34.9%)	
4	183	93 (50.8%)	90 (49.2%)	
4s	53	51 (96.2%)	2 (3.8%)	
**ISSN Stage**				
1,2,3 & 4s	315	276 (87.6%)	39 (12.4%)	0.000
4	183	93 (50.8%)	90 (49.2%)	
**MYCN**				
No amp	401	332 (82.8%)	69 (17.2%)	0.000
Amp	92	33 (35.9%)	59 (64.1%)	
**Risk**				
Low, Intermediate	322	291 (90.4%)	31 (9.6%)	0.000

High	176	78 (44.3%)	98 (55.7%)	
**OS**				
No	393	328 (83.5%)	65 (16.5%)	0.000
Yes	105	41 (39.0%)	64 (61.0%)	
**EFS**				
No	315	268 (85.1%)	47 (14.9%)	0.000
Yes	183	101 (55.2%)	82 (44.8%)	
**FAV**				
Favorable	181	175 (96.7%)	6 (3.3%)	0.000
Unfavorable	91	39 (42.9%)	52 (57.1%)	

EFS: Event-free survival; FAV: Unfavorable/Favorable (class label for extreme disease course); HR: High-risk patients; OS: Overall survival.

Table S6. Neuroblastoma tumor samples clinical data.

Sample	Patient	Age	Risk Group	MYCN Amplification	Clinical Situation at Sample Collection	Treatment sStatus
VH-N01	VH-P01	5 y	Ganglio-neuroblastoma	NA	Resection at diagnose	Pre-treatment
VH-N02	VH-P02	9 m	Low	Unknown	Resection at diagnose	Pre-treatment
VH-N03	VH-P03	1 m	Low	Unknown	Resection at diagnose	Pre-treatment
VH-N04	VH-P04	1 y 8 m	Low	Unknown	Primary tumor resection	Post-treatment
VH-N05	VH-P05	11 m	Low	NA	Resection at diagnose	Pre-treatment
VH-N06	VH-P06	1 y 2 m	Low	NA	Resection at diagnose	Pre-treatment
VH-N07	VH-P07	1 m	Low	NA	Biopsy at diagnose	Pre-treatment
VH-N08	VH-P08	16 m	Low	Unknown	Primary tumor resection	Post-treatment
VH-N09	VH-P09	2 y	Intermediate	NA	Biopsy at diagnose	Pre-treatment
VH-N10	VH-P10	1 y 9 m	Intermediate	NA	Biopsy at diagnose	Pre-treatment
VH-N11	VH-P11	2 y	Intermediate	NA	Primary tumor resection	Post-treatment
VH-N12	VH-P12	2 y	Intermediate	NA	Primary tumor resection	Post-treatment
VH-N13	VH-P13	4 y	High	NA	Primary tumor resection	Post-treatment
VH-N14	VH-P14	7 y	High	Unknown	Primary tumor resection	Post-treatment
VH-N15	VH-P15	4 y	High	A	Primary tumor resection	Post-treatment
VH-N16	VH-P16	5 m	High	A	Primary tumor resection	Post-treatment
VH-N17		20 m			Relapse resection (Cerebellum metastasis)	Post-treatment
VH-N18	VH-P17	2 y	High	Unknown	Primary tumor resection	Post-treatment
VH-N19	VH-P18	2 y	High	NA	Biopsy at diagnose	Pre-treatment
VH-N20		3 y			Relapse resection (Liver metastasis)	Post-treatment
VH-N21	VH-P19	8 y	High	NA	Resection at diagnose	Pre-treatment
VH-N22	VH-P20	11 m	High	A	Primary tumor resection	Post-treatment
VH-N23	VH-P21	2 y	High	A	Primary tumor resection	Post-treatment
VH-N24	VH-P22	3 m	High	A	Biopsy at diagnose	Pre-treatment

Abbreviations: in *Age* column *y* means 'years' and *m* means 'months'; in *MYCN amplification* column *NA* means 'non-amplified' and *A* means 'amplified'.

SUPPLEMENTARY METHODS

Human neuroblastoma samples

Primary tumor tissue samples from 22 neuroblastoma patients enrolled at the Vall d'Hebron Hospital (Barcelona, Spain) were obtained immediately after surgery or biopsy and snap frozen in liquid nitrogen and stored at −80 °C until processing (Registered collection Ref. C.0002311). Tumors were examined by the pathologist to confirm neuroblastoma diagnosis and the presence of at least 80% of tumor tissue sample and histopathologic classification. All patients gave written informed consent.

Therapeutic agents for drug resistance experiments

Therapeutic agent cis-Diammineplatinum(II) dichloride (cisplatin) was purchased from Sigma-Aldrich (St. Louis, MO, USA) and topotecan and 13-cis-retinoic acid (Isotretinoin) were purchased from Selleckchem (Houston, TX, USA).

Immunohistochemistry

Formalin-fixed paraffin-embedded samples from a neuroblastoma patient from Vall d'Hebron Hospital and a healthy kidney were used for immunohistochemistry procedures, following the recommendations described in the Human Protein Atlas, using anti-DNAJC2 (ZRF1) HPA020454 antibody from Sigma-Aldrich (St. Louise, MO, USA), at 1:50 dilution. ZRF1 signal of the different cellular components found on each slide was analyzed by an expert pathologist from Pathology department of Vall d'Hebron Hospital.

Transcriptomic analysis

SK-N-BE(2) cells were seeded at a density of 2.5 x 10^5 cells per well in a 6-well plate and transduced with Non-Silencing Control (NSC), shZRF1 #1 or shZRF1 #3 in technical triplicates. RNA was isolated after 72 hours using the miRNeasy mini kit (Qiagen, Germantown, MA, USA). RNA quality was evaluated using Agilent Bioanalyzer 2100 Eukaryote Total RNA Pico assay (Agilent Technologies, Santa Clara, CA, USA). A total of 300 nanograms of total RNA were hybridized to Human ClariomTM S assay (Affymetrix, Santa Clara, CA, USA) arrays with the GeneChip WT Terminal Labelling and Hybridization Kit (Affymetrix). Differential gene expression analysis was performed using the Transcriptome Analysis Console version 4.0.0.25 (Thermo Fisher Scientific) and functional annotations of resulting gene lists were performed using GSEA software [1,2]. The accession number for microarray analysis reported in this paper is GSE168939.

Cell cycle analysis

Transduced cells were fixed 72 h after transduction in 70% ice-cold ethanol overnight at -20 °C, at a density of 10^6 cells/mL. The fixed cells were washed twice with PBS and resuspended in a working solution containing 15 µg/mL propidium iodide, 1.14 mM sodium citrate, and 0.3 mg/mL RNase A in PBS at a density of 10^6 cells/mL. Cells were incubated at room temperature (20–25 °C) in the working solution for at least 30 min prior to analysis with a FACSCalibur flow cytometer (BD Biosciences).

Subcellular localization analysis

Nuclei of neuroblastoma cell lines were isolated from cytoplasmic fraction by lysing cells in a hypotonic buffer (10 mM Hepes, 10 mM KCl pH 7.9, 1.5 mM $MgCl_2$, protease inhibitors) and applying 50 strokes with a Potter Elvehjem homogenizer. Nuclei were pelleted (2000 xG, 15 min, 4ºC) and cytoplasmic fraction (supernatant) was kept. Nuclei were lysed using RIPA buffer and passing them 5 times through a 25-gauge needle. Cell debris was pelleted and nuclear fraction was kept. A total of 30 µg of cytoplasmic or nuclear fractions were used for western blot analysis, using GAPDH (1:10000 in 5% milk; Santa Cruz Biotechnology, sc-32233) and histone H3 (1:10000 in 5% milk; Millipore, #05-928) as cytoplasmic and nuclear markers, respectively.

REFERENCES:

1. Mootha VK, Lindgren CM, Eriksson KF, Subramanian A, Sihag S, Lehar J, Puigserver P, Carlsson E, Ridderstråle M, Laurila E, Houstis N, Daly MJ, Patterson N, Mesirov JP, Golub TR, Tamayo P, Spiegelman B, Lander ES, Hirschhorn JN, Altshuler D, Groop LC. PGC-1α-responsive genes involved in oxidative phosphorylation are coordinately downregulated in human diabetes. *Nat Genet*. **2003**;*34(3)*:267–73.
2. Subramanian A, Tamayo P, Mootha VK, Mujherjee S, Ebert BL, Gillette MA, Paulovich A, Pomeroy SL, Golub, Todd R, Lander ES, Mesirov JP. Gene set enrichment analysis: A knowledge-based approach for interpreting genome-wide expression profiles. *Proc Natl Acad Sci U S A*. **2005**;*102(43)*:15545–50.
